# Effects of Population Based Screening for Chlamydia Infections in The Netherlands Limited by Declining Participation Rates

**DOI:** 10.1371/journal.pone.0058674

**Published:** 2013-03-20

**Authors:** Boris V. Schmid, Eelco A. B. Over, Ingrid V. F. van den Broek, Eline L. M. Op de Coul, Jan E. A. M. van Bergen, Johan S. A. Fennema, Hannelore M. Götz, Christian J. P. A. Hoebe, G. Ardine de Wit, Marianne A. B. van der Sande, Mirjam E. E. Kretzschmar

**Affiliations:** 1 Center for Infectious Disease Control, RIVM, Bilthoven, The Netherlands; 2 Center for Nutrition, Prevention and Health Services, RIVM, Bilthoven, The Netherlands; 3 STI AIDS Netherlands, Amsterdam, The Netherlands; 4 Department of General Practice, Amsterdam Medical Center/University of Amsterdam, Amsterdam, The Netherlands; 5 Public Health Service Amsterdam, Amsterdam, The Netherlands; 6 Public Health Service Rotterdam Rijnmond, Rotterdam, The Netherlands; 7 Department of Public Health, Erasmus Medical Center, Rotterdam, The Netherlands; 8 Public Health Service Southern Limburg, The Netherlands; 9 School of Public Health and Primary Care (CAPHRI), Maastricht University Medical Centre (MUMC+), Maastricht, The Netherlands; 10 Julius Center for Health Sciences and Primary Care, University Medical Center Utrecht, Utrecht, The Netherlands; Melbourne School of Population Health, Australia

## Abstract

**Background:**

A large trial to investigate the effectiveness of population based screening for chlamydia infections was conducted in the Netherlands in 2008–2012. The trial was register based and consisted of four rounds of screening of women and men in the age groups 16–29 years in three regions in the Netherlands. Data were collected on participation rates and positivity rates per round. A modeling study was conducted to project screening effects for various screening strategies into the future.

**Methods and Findings:**

We used a stochastic network simulation model incorporating partnership formation and dissolution, aging and a sexual life course perspective. Trends in baseline rates of chlamydia testing and treatment were used to describe the epidemiological situation before the start of the screening program. Data on participation rates was used to describe screening uptake in rural and urban areas. Simulations were used to project the effectiveness of screening on chlamydia prevalence for a time period of 10 years. In addition, we tested alternative screening strategies, such as including only women, targeting different age groups, and biennial screening. Screening reduced prevalence by about 1% in the first two screening rounds and leveled off after that. Extrapolating observed participation rates into the future indicated very low participation in the long run. Alternative strategies only marginally changed the effectiveness of screening. Higher participation rates as originally foreseen in the program would have succeeded in reducing chlamydia prevalence to very low levels in the long run.

**Conclusions:**

Decreasing participation rates over time profoundly impact the effectiveness of population based screening for chlamydia infections. Using data from several consecutive rounds of screening in a simulation model enabled us to assess the future effectiveness of screening on prevalence. If participation rates cannot be kept at a sufficient level, the effectiveness of screening on prevalence will remain limited.

## Introduction

Chlamydia infections are the most prevalent bacterial sexually transmitted infection (STI) in the Netherlands with a prevalence of around 2% in the adult population [1], [2]. Prevalence is highest in younger age groups and in urbanized areas. As Chlamydia infections in women can cause long term complications such as pelvic inflammatory disease (PID), ectopic pregnancy and infertility [3], prevention is needed especially in young women. Around 80% of infections in women and 50% of infections in men are asymptomatic [4], implying identification of asymptomatic infections is key for a successful control of the infection.

In the Netherlands, the implementation of a national Chlamydia screening program has been in discussion for many years [5]. Pilot studies were conducted to investigate the feasibility of opportunistic and population based screening, respectively [1], [6], and cost effectiveness studies suggested that screening could be cost-effective or even cost saving if implemented in certain age groups [7]–[9]. A study to obtain baseline estimates of prevalence in the general population was conducted in four regions in the Netherlands, covering both urban and rural population [1]. To support a final decision about implementation of a national Chlamydia screening program a register-based screening implementation (Chlamydia Screening Implementation) was piloted in three regions in the Netherlands in 2008–2012 [10]–[13].

To assess the long term effects of the Chlamydia Screening Implementation (CSI) a dynamic network simulation model was employed that took into account data on sexual behavior in the Netherlands, uptake of chlamydia testing and treatment in regular health care, and participation rates of screening as observed in the CSI. Projections about future incidence of Chlamydia infections in the heterosexual population were combined with a cost-effectiveness study to estimate the cost per major outcome averted.

Here we report on results from the modeling study on effectiveness of population based screening during the CSI and projected ten years into the future. We investigated how the impact on prevalence depends on participation rates and on degree of urbanization. Finally we investigated the effects of alternative screening strategies such as changes in screening intervals and targeted age groups on population prevalence of Chlamydia infection.

## Methods

### A dynamic sexual network model

We used a simulation model that describes a dynamic process of partnership formation and dissolution in a heterosexual population [14]. The population consisted of 50,000 individuals with a sex ratio of 1∶1 and a uniform distribution over the age range 13–64 years. Individuals were characterized by their sex, age, partnership status and infection status. Sexual activity of an individual could change during an individual's life course with periods of high and low rates of partner change. In periods of high sexual activity, the capacity of an individual to form partnerships and maintain them simultaneously was higher than in periods of low activity. In the latter periods, individuals were restricted to monogamous partnerships of varying duration. Partnerships could be of three types distinguished by their average durations and frequencies of sexual intercourse. Sexual behavior parameters were fitted to data about cumulative numbers of life time partners, age at sexual debut, numbers of partners in last year by age and sex, durations of partnerships and gaps between partnerships. The model population is not stratified by ethnicity, neighborhood or educational level. For details about model structure and fitting of those variables we refer the reader to Schmid & Kretzschmar 2012 [14]. Parameter values used by the model are given in Table S1 in Supplementary Information S1.

### Sexual behavior data

We used data from a representative survey on sexual behavior in the age group 15–70 conducted by Rutgers-WPF in 2008 in the Netherlands [15]. The survey included 5402 respondents, 2499 males and 2903 females. We used the following variables to inform the model: numbers of life time partners, numbers of partners in the last 6 months, duration of current partnership, overlap between partnerships with a new(<6 months) steady partner and previous partners, degree of urbanization, age at first sex, and age of partner. All variables were stratified by age and sex. We used information from all respondents to parametrize sexual behavior on the national level (in the following referred to as the **national level model**), and data from those respondents living in highly urbanized areas (>2500 addresses per km^2^) to parametrize a model for the urban areas (referred to as the **urbanized areas model**).

Some additional variables were used to inform the sexual network model for which no information was available in the Rutgers-WPF survey: these are the duration of the previous partnership, the gap/overlap between the last two partnerships, and the degree of assortative mixing between individuals of different levels of sexual activity. For these variables we used the information available in the NATSAL sexual behavior survey, which included 11,161 respondents from the United Kingdom in 2000 [16]. We found the use of sexual behavior data from the UK justified, because we found that both surveys showed reasonably similar results for variables that were available in both data sets (such as the cumulative numbers of life time partners), and that the sexual network model could be adjusted to accommodate these additional variables without deteriorating its fit to the information available in the Rutgers-WPF survey [14].

### Chlamydia transmission

Transmission of Chlamydia is implemented within the model as the daily chance to transmit Chlamydia between an infected and an uninfected partner. The Chlamydia transmission rate was chosen such that the prevalence in both the national and urban sexual network models matched the Chlamydia prevalence reported for different urbanization degrees by van Bergen et al [1]. The duration of untreated Chlamydia, and the incubation time (which also defined the latent period) were taken from [17], [18], and are listed in Table S1 in Supplementary Information S1. The fraction of infections that was symptomatic was set to 50% for men, and 30% for women, based on the experience of an expert panel composed of GPs and members of the Dutch STI centers.

### Chlamydia testing and treatment in regular health care

In the Netherlands, Chlamydia infections are diagnosed either by general practitioners (GPs) or at STI clinics. The latter serve the high risk population for sexually transmitted infections, whereas the former diagnose infections also in the general population. If a Chlamydia infection is diagnosed, antibiotic treatment is given and an effort is made to notify and treat current and ex-partners. Chlamydia infections are not notifiable in the Netherlands, but a sentinel surveillance based on STI clinic diagnoses is used to monitor trends in prevalence. In recent years, an increasing trend was observed in the numbers of consultations for STI testing at STI clinics, however positivity rates remained fairly stable [19]. A substantial fraction of cases are diagnosed and treated by GPs and are not reported to national surveillance [20]. In 2001, Chlamydia prevalence was measured in the general population in a sample of 3458 men and 4925 women [1]. Prevalence was 2.5% among women and 1.5% among men, with higher prevalence in younger age groups and a clear association of high prevalence with degree of urbanization.

In the model, we assumed that there is a group of 9% of the population that never seeks health care for Chlamydia infections even when they are symptomatic [21]. This population group is also not compliant with requests from their partners to seek treatment in the context of partner notification. For the remaining population, individuals with symptomatic infections will seek healthcare. We assumed that symptoms develop after an incubation period of 14 days, and that individuals visit a GP after appearance of symptoms with a median delay of 16 days (sampled from an exponential distribution). In the model, symptomatic men receive treatment and clear the infection immediately after their visit to the GP. For symptomatic women, it takes an additional 8 days after visiting a GP until there is laboratory confirmation of a positive Chlamydia test, after which they immediately receive treatment and clear the infection [22]. Individuals with asymptomatic infections seek healthcare with a rate estimated from data about GP and STI center consultations [19]. If they are diagnosed positive for Chlamydia, they receive treatment and clear the infection immediately. When in the model a Chlamydia infection is diagnosed by the GP or laboratory, an average of 40% of current partners are treated for Chlamydia as well [23]; in the model partners are treated on the same day as the infected index case. We assumed a Chlamydia test sensitivity of 98%, a Chlamydia test specificity of 99.6% [24], and a treatment success rate of 97% [25]. The testing and treatment of symptomatic and asymptomatic Chlamydia infections as described here forms the **baseline healthcare level** against which the effect of the screening implementation is tested. See also table 1 for a summary of assumptions regarding screening and treatment.

**Table 1:** Screening rules implemented in the model.

Screening design	Rule as implemented in the model	Comments	Reference
1 Time of invitation	Each individual is assigned a number which indicates the week of the year that they are invited for CSI screening.		
2 Eligibility	Individuals are eligible to participate if they are in the age group 16–29 years and meet the criteria for inclusion (e.g. have been sexually active, and for the national level model, pass a risk-score threshold to exclude those persons with negligible risk levels).	In the model, individuals who turn 16 during the year are invited for the first time in the following year; this might be slightly different in reality, where they might still be invited the same year they turn 16, depending on the exact timing of invitations per geographic area.	
3 Risk score selection	The risk score is explained in table 2. It excludes about 20% of the lower-risk sexually active population in the age-range of 16–29.	The risk score is similar to the risk score-based selection applied in one of the CSI regions. As the model population was not stratified by level of education or ethnicity, the risk score threshold for inclusion used in the model was lower than the > = 6 threshold used in the CSI to exclude a similar fraction of 20–30% of the population as was excluded by risk score in the CSI screening.	[26] [13]
4 Acceptance of first invitation	The chance that an individual accepts his/her first screening invitation depends solely on their gender, and the number of years since start of the screening program Initial participation decreases for both genders over time.	Factors for decrease of initial participation over time are 2008: 1.0 2009: 0.82 2010: 0.675 2011: 0.438 2012 and further: 0.37 times participation in 2008.	
5 Repeated acceptance	Repeated participation depends solely on an individual's previous decisions on participation. The chance to participate per screening round is detailed in figure 1.		
6 Treatment uptake	14% of participants ignore positive test results, and do not seek treatment. In the model there is no correlation between these 14% and the 9% of the population that do not participate in baseline healthcare. 86% of those tested positive get treatment.	In the CSI 91% sought treatment after being informed positive, 94% of those 91% actually took the treatment.	[39], page 46
7 Treatment of current partners	80% of current partners of individuals treated for Chlamydia are notified and treated at the same time as the index case. The 20% of the current partners that are not treated includes the 14% that would ignore their own positive test results (point 6), and individuals who have been tested or treated themselves recently (a personal value for each individual, drawn from an exponential distribution with a median of 68 days).	“recent testing/treatment fatigue” determines whether individuals are willing to participate in testing and/or treatment as part of symptomatic and asymptomatic regular healthcare, as well as part of all forms of partner notification (both regular healthcare and CSI), for a number of days after their latest Chlamydia testing and/or treatment. Participation in the CSI program in the model is not affected by this fatigue, as the participation data upon which the participation trees are based already implicitly contains this information (on a population level)	[40]
8 treatment of ex-partners	50% of ex-partners for which the partnership ended less than one year ago are notified and treated. The 50% of the recent ex-partners that are not treated include the 14% that would ignore their own positive test results, as well as those that have been treated recently. As a model simplification, treatment of ex-partners happens immediately upon treating the positively screened individual.		
9 Retesting of those tested positive	In the case of a positive test result, participants are invited for an additional test 6 months after the initial invitation. The procedure is identical to the above procedure, except that the delay between invitation and treatment is shorter by 17 days. The effect is that people are re-treated (if positive) 166 days after their first test.	In the CSI these participants immediately get a test-kit sent to their home, which shortens the delay between an invitation to be retested and actual treatment taking place by 17 days.	

### Chlamydia Screening Implementation data

The CSI was carried out in three regions in the Netherlands, in which different screening procedures were set up in order to be able to evaluate the impact of screening on prevalence after 3 subsequent screening rounds. In the CSI, both men and women in the age group 15–29 yearly received an invitation to participate in screening. Invitations were personalized letters including the address of the program website and a secure login code through which eligible participants could request a kit for self-sampling. In the less urbanized areas, the expected chlamydia prevalence was lower than in the big cities and therefore, a selection through risk score based on data from a prior pilot chlamydia screening project was used. Scores were based on age, urban/non-urban residence, level of education, ethnic background, condom use, number of lifetime and recent sex partners, and presence of STI-related symptoms, as reported in a short online questionnaire to be answered before ordering a test kit [11], [26]. For more detail, we refer the reader to [10]–[12]. Tables 1 and 2 describe how those procedures were defined in the simulation model. Table 3 shows numbers of participants per screening year. Partner notification rates were around 80% in the CSI and therefore higher than observed in earlier GP based studies.

**Table 2:** Risk score calculation.

Variable	Risk Score
Age less than or equal to 19	+1, else +0
Lifetime partners 1	+0
Lifetime partners 2–5,	+2 for men, +3 for women
Lifetime partners 6+	+3 for men, +5 for women
New relationship in last 6 months?	+1, else +0
Cutoff value for men:	20% has a risk-score<2, and is excluded from screening
Cutoff value for women:	18% has a risk-score<3, and is excluded from screening

**Table 3:** The numbers of men and women, who participated in the CSI per year of screening.

	Men yes	Men no	participation rate (%)	Women Yes	Women no	participation rate (%)
2008	13176	109847	10.7	28853	104932	21.6
2009	10625	138285	7.1	24201	135088	15.2
2010	8722	144721	5.7	20610	142234	12.7
2011	1152	44135	2.5	2962	43159	6.4

The total numbers of men and women differ between years, mainly because both in 2008 and in 2011 the CSI program did not run during the whole year.

Approval for this study was obtained from the Medical Ethics Committee of the Free University of Amsterdam. The study is also registered in the NTR 3071 (Netherlands Trial Register, www.trialregister.nl). Informed consent was given by application for a test kit in writing via an online project website, and this consent procedure was approved by the ethics committee.

### Participation trees

**Figure 1:**
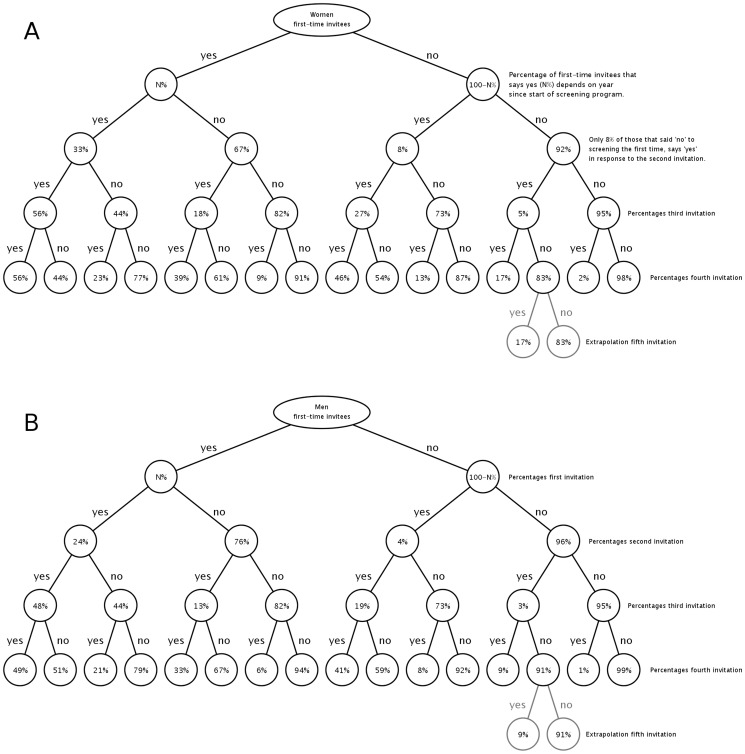
Participation trees for women and men. First-time participation was modelled to depend only on gender (panel A for women, panel B for men), and year since the start of the screening program. Subsequent participation depended solely on the previous choices made. All rates are based on observed participation rates in subsequent rounds of CSI. The extrapolation for years after the 4 years of CSI is based on results of the fourth round as described in the text.

We describe changes over time in participation rates based on a so-called participation tree, i.e. a scheme that defines the probability of an individual to participate in screening depending on his/her previous participation decision (Figure 1). If an individual is offered screening for the first time, he/she participates with a probability that depends on gender and year since the roll-out of the screening program. Factors describing decline of initial participation from one year to the next are given in Table 1. In subsequent screening rounds, the individual gets a new offer and now the probability to participate depends on the history of participation decisions of that individual. After 4 screening rounds each participant can be characterized by a sequence of YES and NO describing whether or not he/she participated in a particular round. We stratified the data set according to unique sequences of YES and NO. From these sub data sets the percentages in the participation trees were computed. The same data was used to validate the assumption that subsequent screening rounds only depend on the previous choices that an individual has made. This was done by comparing the participation trees based only on participation decisions of individuals starting CSI in 2008 with those of individuals starting in later years.

Participation trees are extrapolated beyond the fourth round (the last round for which we have CSI participation information) by assuming that in the fifth and subsequent rounds, the ratio of participants and non-participants remains stable, that is remains equal to the ratio of participants and non-participants observed in the fourth round in that branch of the tree. As an example, in Figure 1 the extrapolation is shown for one particular branch of the tree, which shows how the chances of accepting and declining a fifth invitation are the same as for the fourth invitation (i.e. 17% and 83%). This is the simplest extrapolation possible; although more sophisticated ways of extrapolating participation can be defined based on the participation data, the impact on the model results using such extrapolations was found to be very small.

### Screening scenarios

#### National level model versus urbanized areas model

We considered two types of populations, one that represented the general population on the national level, and another one that represented urbanized areas only. For the model on the national level, the data from the complete RUTGERS-WPF sexual behavior survey was used and it was assumed that there was a selection for screening based on risk scores. For the model describing urbanized areas, we used sexual behavior data only from individuals from highly urbanized areas and there was no risk score selection.

#### CSI screening scenario

The CSI screening scenario describes screening as implemented in the CSI project [11], [12], [27]. In short: in the CSI, both men and women in the age group 15–29 are assigned a particular week number, in which they yearly receive an invitation to participate in screening. The time between invitation and actual treatment of those found positive depends on the time that it takes individuals to respond to their invitation, get a sampling kit, return the sample, and if tested positive, acquire treatment [28]. Participation of individuals in the simulation model was implemented as described above, with the additional assumption that all the described delays are constant over time. This allowed us to assign a week number to every individual in the model that reflects their invitation week and personal delay until treatment. Individuals who tested positive, complied with treatment with probability 0.86, and were treated immediately (Table 1).

#### Alternative screening scenarios

We considered four alternative screening scenarios:

- **Screening women only**
  As the long-term harmful effects of Chlamydia almost exclusively affect women, screening women only (and their partners after positive initial test results) may be a more cost effective strategy.
- **Screening the age group 16–24**
  As Chlamydia prevalence is generally thought to be highest in the age range of 16–24, targeting the screening to that age-group may be more cost-effective. Screening programs in other countries indeed target to the age range 16–24 years [29].
- **Screening the age group 26–29**
  Peak prevalence in men is in older age groups, so targeting those age groups may also have considerable effects on younger women.
- **Biennial screening**
  Screening individuals once every two years rather than annually may prevent participation exhaustion by inviting participants less frequently. Participation retention in the biennial screening scenario is handled as follows: only half the eligible CSI group participates gets their first invite in 2008, the other half gets their first invite in 2009. First time participation is still determined by gender and year since introduction of the CSI program, so the group that gets their first invite in 2009 will have the first-time participation ratio associated with 2009 (as in the other CSI simulations). The time between invitations per individual is now 2 years, rather than 1 year. This is assumed to have no effect as to how individuals move through the participation trees (as we have no data from which to estimate how participation trees might change if invitations are send out once every two years).

As all four scenarios reduce the size of the target population of the screening program as compared with the CSI screening, their effects on Chlamydia prevalence will inevitably be less than that of the CSI strategy. However, cost effectiveness of those alternative strategies may more favorable.

#### Sensitivity analysis

Besides those alternative scenarios we included two scenarios into our results that investigated sensitivity of results on assumptions and model input. One is a scenario, where we assumed a stable high participation rate for both men and women. We included this to show how prevalence would have developed if the CSI would have been more successful in recruiting participants into screening. The CSI was set up with the expectation that the participation rates of around 30% found in the 1-year pilot study [1] could be maintained. This proved to be wrong on two accounts: the fraction of initial invitees that participated declined after the first year, and persons that were invited a second and third time on average participated less than they did in response to the previous invitations. On the national level, we study the effect of an average 30% participation rate, split into 19.9% for men, and 40.1% for women (the same relative difference in participation rates between genders as observed in CSI). On the urban level, we studied the effect of an average 25.6% participation rate (i.e. the same relative difference in participation rates between the national and urban level as was observed during CSI). Next, we also consider the possibility of high levels of treatment failure, which by some estimates could be as high as 8% [30], [31]. In this scenario we assumed that the high level of treatment failure occurred both in regular healthcare and in CSI, and we re-fitted the daily Chlamydia transmission chance of the model, such that the baseline Chlamydia prevalence in the high treatment failure scenario remains similar to the baseline Chlamydia prevalence in the main model. We compared the impact of CSI with and without high levels of treatment failure.

### Model implementation

For every scenario 40 simulation runs were conducted in a population of 50,000 individuals and prevalence was averaged over those simulation runs. To quantify the impact of screening in the long run we compared with a baseline scenario in which no screening was implemented. In the baseline scenario, Chlamydia prevalence dropped slightly over time due to the increase of Chlamydia testing and treatment in regular care as extrapolated from observed trends [19]. We analyzed variability of results across runs. We also performed simulations with smaller and larger population sizes to ensure that the chosen population size was sufficiently large to deliver stable results (not shown).

## Results

**Figure 2:**
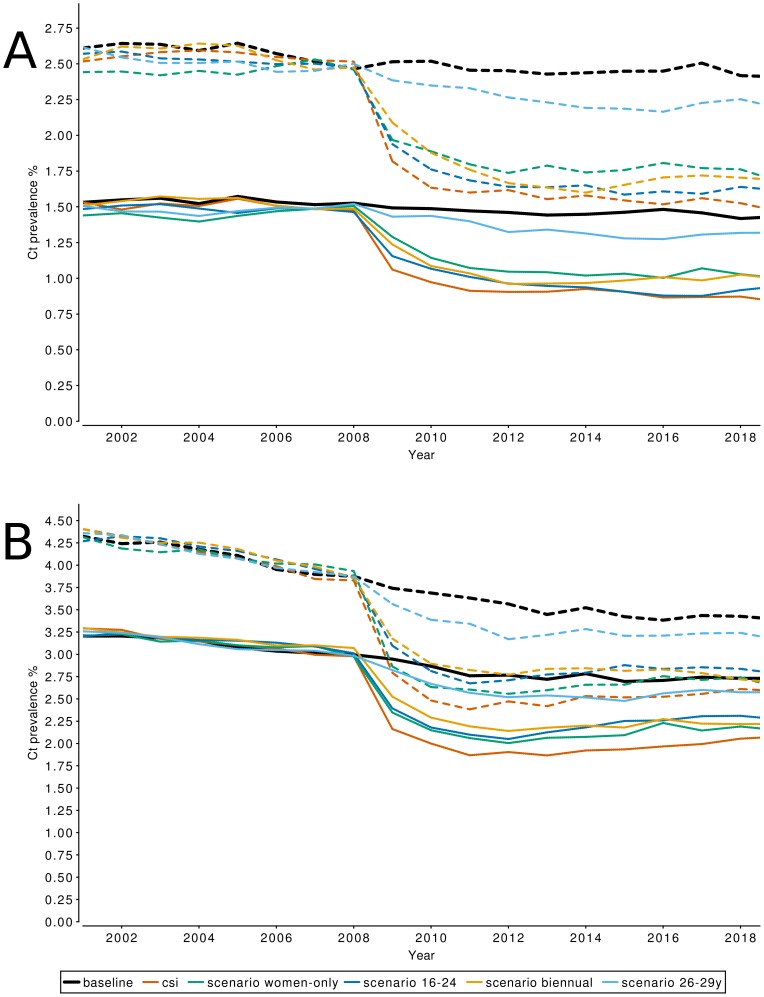
Effect of CSI on population prevalence of Chlamydia infections. The projected Chlamydia prevalence for men (solid lines) and women (dashed lines) for the baseline scenario, CSI screening, and alternative scenarios. Panel A shows the projected prevalence on the national level, Panel B shows the projected prevalence in urbanized areas.

**Figure 3:**
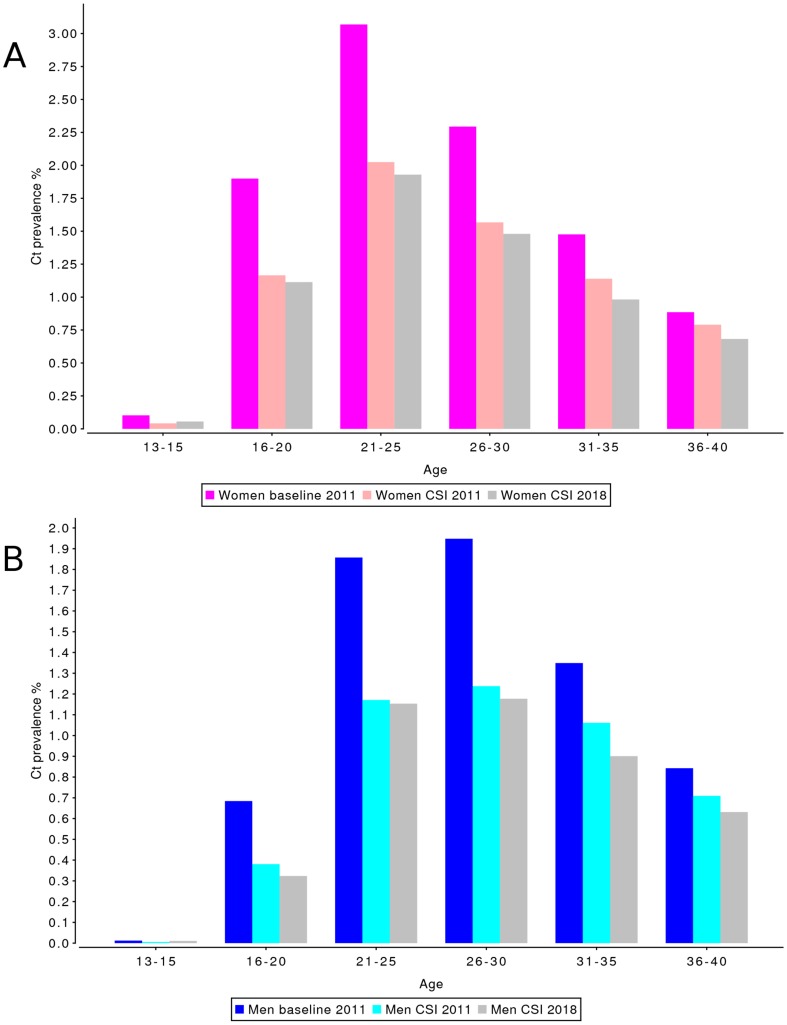
Effect of CSI on population prevalence of Chlamydia infections by age category, on the national level. Prevalence levels are shown for a scenario without screening implementation (“baseline 2011”), and after 3 (“CSI 2011”) and 10 years (“CSI 2018”) of screening, for (A) women and (B) men. Splitting the population into age-groups gives a detailed view on the effect of CSI screening in addition to baseline testing and treatment at GPs and STD clinics.

For the CSI, the simulated national level prevalence at the moment of implementation of screening was 2.5% in women and 1.5% in men (Table 4). Prevalence dropped by 0.70 percentage point (pp) in women and 0.41pp in men in the first year of screening. After 3 years of screening, Chlamydia prevalence was 1.6% for women and 0.9% for men, i.e. a decrease in prevalence by 0.92pp for women and 0.56pp for men as compared to prevalence at start of screening, and a decrease of 0.86pp for women and 0.56pp for men compared to the Chlamydia prevalence in the baseline scenario at the beginning of 2011. From 2011 onward, the additional effect of screening on Chlamydia prevalence compared to the baseline scenario remained constant (Figure 2A). The absolute impact of screening was largest in the age groups 21–29 years for both men and women and lower for age groups outside of the targeted age range for both men and women (Figure 3).

**Table 4:** Summary of impact of screening on population prevalence of Chlamydia infections as computed by the model.

		*Chlamydia prevalence*				*Decline in Chlamydia prevalence*	
		at start of screening	after 1 year of screening	after 3 years of screening	after 10 years of screening	after 3 years of screening compared with prevalence at start of screening	after 10 years of screening compared to no screening
year		2008	2009	2011	2018		
national	women	2.5%	1.8%	1.6%	1.5%	0.92pp	0.89pp
	men	1.5%	1.1%	0.9%	0,9%	0.56pp	0.55pp
urban	women	3.8%	2.8%	2.4%	2.6%	1.45pp	0.82pp
	men	3.0%	2.2%	1.9%	2.1%	1.11pp	0.68pp

Declines are measures in percentage points (pp), i.e. the difference between two percentages. For comparison, the prevalence estimates derived from the positivity measures in the CSI program [13] are presented in Table 5.

**Figure 4:**
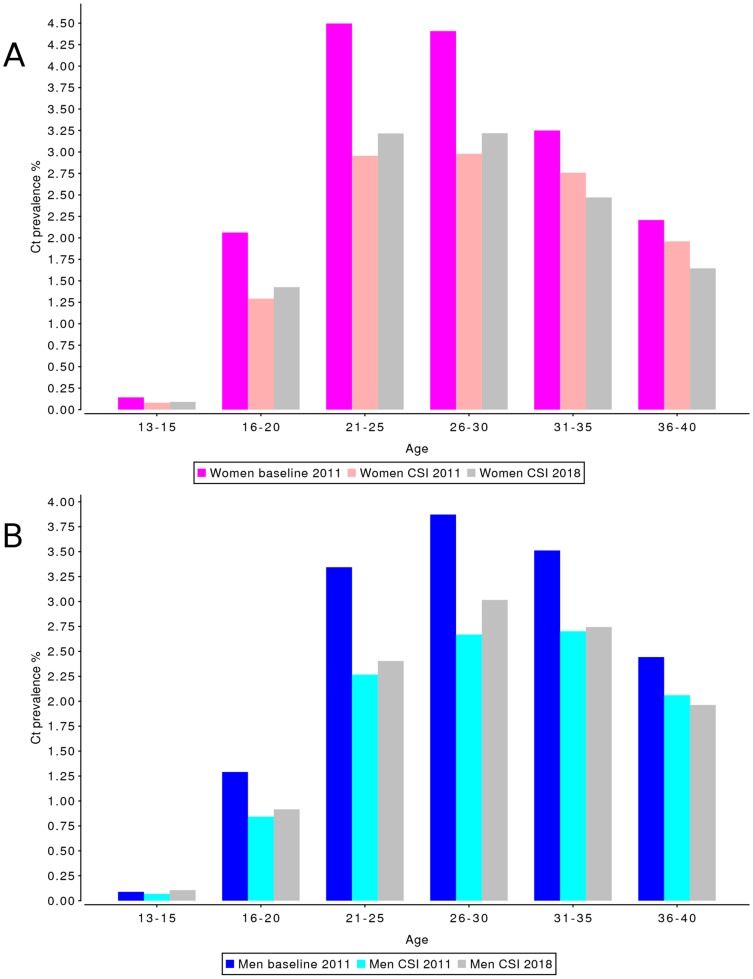
Effect of CSI on population prevalence of Chlamydia infections by age category, in urbanized areas. Prevalence levels are shown for a scenario without screening implementation (“baseline 2011”), and after 3 (“CSI 2011”) and 10 years (“CSI 2018”) of screening, for (A) women and (B) men. The effect on Chlamydia prevalence is most visible in the age-groups 21–25 for both women and men.

For the CSI screening scenario in the highly urbanized areas, Chlamydia prevalence at the time of implementation of the screening program was 3.8% in women and 3.0% in men (Table 4). Prevalence then dropped by 1.04pp in the first year of screening in women, and by 0.82pp in men. Chlamydia prevalence in the screening scenario reached a minimum of 2.4% in women and 1.9% in men at the beginning of 2011, but then increased again until the beginning of 2018, where it reaches a level of 2.6% for women and 2.1% for men. The Chlamydia prevalence for the CSI screening scenario in 2018 is 1.22pp lower for women, and 0.92pp lower for men, compared to the Chlamydia prevalence at the start of screening at the beginning of 2008; however, due to a predicted continuing decline in Chlamydia prevalence in the urban baseline scenario the prevalence in 2018 is only 0.82pp lower for women, and 0.68pp lower for men compared to the baseline scenario Chlamydia prevalence in 2018 (Figure 2B). Again the absolute impact of screening was largest in the age groups 21–29 years for both men and women and lower for age groups outside of the targeted age range for both men and women (Figure 4). For the 16–20 year old women the impact of screening was larger on national level than in the urbanized areas.

For comparison, the prevalence estimates derived from the positivity measures in the CSI program [13] are presented in Table 5.

**Table 5:** Prevalence estimates derived from positivity measures in the CSI program.

		Chlamydia prevalence at first invitation	Chlamydia prevalence at second invitation	Chlamydia prevalence at third invitation
year		2008	2009	2010
Limburg	women	3.00%	2.80%	2.00%
	men	2.40%	2.10%	1.30%
Amsterdam	women	2.73%	2.68%	2.63%
	men	2.50%	2.30%	2.31%
Rotterdam	women	4.03%	3.61%	4.08%
	men	3.33%	3.32%	3.81%

		Chlamydia prevalence at first invitation	Chlamydia prevalence at second invitation	Chlamydia prevalence at third invitation
national	women	3.00%	2.80%	2.00%
	men	2.40%	2.10%	1.30%
urban	women	3.38%	3.15%	3.36%
	men	2.92%	2.81%	3.06%

Prevalence rates for Amsterdam and Rotterdam are aggregated to come to an estimate for urban populations, while estimates for the Limburg area were used as national level estimates. Estimates are only available for 2008–2010 [13].

### Alternative scenarios

#### Screening women only

Despite the high success rate of current partner notification (80%) in the CSI program, screening women only is the least effective alternative scenario, reaching only 50% of the decrease in prevalence that screening both men and women achieves (Figure 2). This result is in contrast with an earlier analysis [32], in which screening of men and women had little additional value above screening women only. A key difference between [32] and the present study is that the former had a high and constant yearly participation rate of women in the age-range 15–24.

#### Screening the age group 16–24 years

The initial decrease in Chlamydia prevalence in the age group 16–29 years is smaller when targeting to 16–24 year olds than in the CSI scenario, because those of age 25–29 were not invited to participate. However, on the national level the difference in prevalence reduction when screening ages up to 24 or up to 29 becomes marginal after 6 years of screening (Figure 2A). In urbanized areas, the 16–24 scenario appears to be permanently less effective than the CSI screening scenario (Figure 2B).

Although the peak of Chlamydia prevalence in women in urbanized areas falls between the ages of 16 and 24, for men living in an urban environment, and for both men and women in a non-urban environment, the peak prevalence is at age 26–29 (Figures 3 and 4). Therefore, when the aim of the program is to maximize the reduction in Chlamydia prevalence, the CSI age range of 16–29 would appear to be the best age range for a nationwide screening program in the Netherlands.

#### Screening the age group 26–29 years

Targeting this older age group only is clearly much less effective than all other scenarios. Apparently, even if peaks in prevalence are observed in those age groups, they are not the groups who are mainly driving the transmission. The reason may be that persons in this age group are recipients of infection, but not responsible for generating many new secondary cases.

#### Biennial Screening

This scenario causes the largest decrease in Chlamydia prevalence of the tested alternative scenarios at short term, but does not lead to a lower Chlamydia prevalence than the CSI screening scenario in the long run. One assumption in the biennial scenario is that participation rates are not affected by the time interval between CSI invites, but only by the number of invitations that an individual gets. As such, it takes longer for individuals to become exhausted with CSI participation, and the average number of years after which individuals permanently stop participating doubles from 4.5 years to 9 years in urbanized areas. A consequence of spreading participation over a longer time period is shifting the age at which individuals participate to correspond with the peak in Chlamydia prevalence, thus increasing the effectiveness of screening.

### Sensitivity analysis

**Figure 5:**
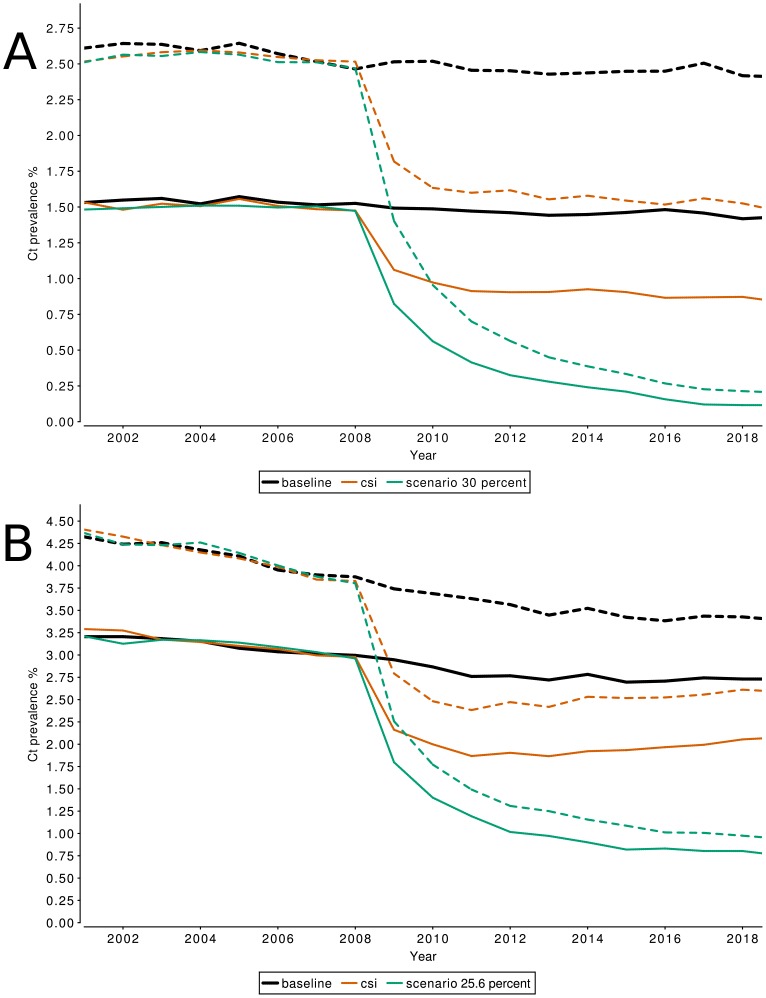
Effect of a high CSI participation rates on population prevalence of Chlamydia infections. In contrast to the reduction in Chlamydia prevalence achieved by screening with the observed participation rates, CSI screening with a stable participation rate of 30% on national level (A) and 25.6% in urbanized areas (B) would lead to a drastic reduction in Chlamydia prevalence in men (solid lines) and women (dashed lines). On the national level, closed populations of 50,000 individuals are frequently unable to maintain Chlamydia in the population, and the average Chlamydia prevalence reported in panel A is therefore a combination of simulated populations where Chlamydia has gone extinct, and where Chlamydia is maintained at low prevalence levels.

The scenario with a high screening participation of around 30% showed that prevalence can be reduced substantially in the long run if participation on that level can be achieved (Figure 5). The prevalence levels reached after 10 years of screening were 0.21% in women and 0.12% in men. The scenario considering high levels of treatment failure showed only marginal impact of that parameter on the effects of screening in the long run (Figure S1 in Supplementary Information S1).

All results shown here are averages over 40 simulations with 50,000 individuals each. Despite these large samples, the estimated average Chlamydia prevalence for the CSI age range showed around 0.1pp variation over time in a population with a Chlamydia prevalence of 1% and 2%, respectively. There was considerable impact of stochasticity on prevalence per simulation which was inherent to the transmission dynamics in the modeled system: sexual contact networks follow a power-law distribution [14], [18], [33], meaning that regardless of population size, there are always a small number of individuals that are considerably more sexually active than the majority of the population and will therefore disproportionally impact on prevalence.

## Discussion

In this modeling study we estimated that the effects of the CSI program as implemented in 2008–2011 in three regions in the Netherlands reduced Chlamydia prevalence in women aged 16–29 by 1.18pp compared to the Chlamydia prevalence prior to the start of the program, and 1.11pp compared to the baseline Chlamydia prevalence in 2011. It is expected that this effect on prevalence will shrink to 0.85pp if the screening program is continued in future years due to a continued decline in participation rates. The effectiveness of the screening program as implemented now is limited by low participation rates and if participation rates cannot be increased, a further reduction of Chlamydia prevalence cannot be expected. The average size of the effect of CSI on Chlamydia prevalence after 10 years of screening for both genders combined is a decrease of 0.72pp in the general population, and 0.75pp in highly urban areas. These results concur with observations on positivity in CSI-data, especially for non-urban settings, however, the observed decline in the empirical data was smaller than in the model and was not statistically significant [13]. If participation rates of 30% would have been achieved as was originally foreseen for the CSI program, a substantial impact on chlamydia prevalence would be possible in the long run with a reduction on the national level of 2.25pp in women and 1.36pp in men by the year 2018. The CSI study was unique in that it collected participation data in several sequential screening rounds in the same population. Using this information for describing the development of participation rates over time in the model enabled us to estimate the impact of declining participation rates on the effectiveness of screening and to project those effects on chlamydia prevalence to 2012–2018. The low and waning participation levels limit the present and future effectiveness of screening for all intervention strategies we investigated. If continued participation cannot be ensured especially in high risk groups prevalence reduction will remain marginal in the long run. Secondly, the structure of the sexual contact network affects the predicted effectiveness of CSI; the sexual contact patterns in the model determine how partner change rates are distributed over the population. This distribution in turn affects the age range in which Chlamydia prevalence peaks and the speed with which Chlamydia is transmitted through the network.

The model used here extends earlier models [32], [34] by including a more detailed description of sexual behavior parameters [14] and of the uptake of testing and treatment of Chlamydia infections by regular health care. These improvements potentially allow a more accurate projection of the effects of population based screening on Chlamydia prevalence. Some modeling assumptions are still simplified, such as the assumption that treatment leads to clearance immediately, and that partners are treated simultaneously with their index case. These assumptions are optimistic thereby making treatment and partner notification more effective than more conservative assumptions would have done. As those assumptions apply both to baseline testing and to screening scenarios, they only have marginal influence on the comparison of both. This is also confirmed by the minimal impact that higher treatment failure rates have on screening success.

Earlier modeling results predicted a much larger impact of population based screening on Chlamydia prevalence [32], [35], [36] than shown here. The main reason for this discrepancy is that in earlier studies screening was implemented in a baseline situation, in which only symptomatically infected individuals were assumed to be tested and treated and partner notification was not performed. In the present model, screening was implemented on top of a baseline level of testing and treating also asymptomatic cases and a baseline rate of partner notification. In other words, health care in the present situation in the Netherlands already detects and treats substantial numbers of asymptomatic Chlamydia infections, so that additional population screening has a much lower incremental effect on prevalence. Other recent modeling studies investigating the question of how population based screening performs incremental to existing care [37], or what the effects are of improving performance of existing screening programs [38] also found low impact of population based screening on Chlamydia prevalence.

The two main conclusions to be drawn from modeling the impact of CSI on Chlamydia prevalence are that a continuous CSI screening effort will have a limited but stable effect on Chlamydia prevalence, and that the size of this effect depends heavily on the sustained participation rate of a screening program. As our efforts to increase participation rates in the general population were not successful, we need to focus prevention efforts on individuals with highest risk by retesting those found positive and by intensifying partner notification.

## Supporting Information

Supplementary Information S1 Parameter values table and additional figures.
(PDF)
