# Supplementary material for: Effects of Population Based Screening for Chlamydia Infections in The Netherlands Limited by Declining Participation Rates
Source: PLoS One. 2013 Mar 20;8(3):e58674. doi: 10.1371/journal.pone.0058674 (PMC3604006; doi:10.1371/journal.pone.0058674)
Supplement: Supplementary Information S1 — Parameter values table and additional figures. (PDF) [file pone.0058674.s001.pdf]

Supplementary Information File

# Effects of population based screening for Chlamydia infections in the Netherlands limited by declining participation rates

Boris V. Schmid[1, 2], Eelco A.B. Over[3], Ingrid V.F. van den Broek[1], Eline L.M. Op de Coul[1], Jan E.A.M. van Bergen[4,5,1], Johan S.A. Fennema[6], Hannelore M Götz[7,8], Christian J.P.A. Hoebe[9,10] , G. Ardine de Wit[3,11], Marianne A.B. van der Sande[1,11], Mirjam E.E. Kretzschmar[1,11]*

*corresponding author

1 Center for Infectious Disease Control, RIVM, Bilthoven, The Netherlands
2 Present address: Department of Biosciences, Centre for Ecological and Evolutionary Synthesis (CEES), University of Oslo, Norway
3 Center for Nutrition, Prevention and Health Services, RIVM, Bilthoven, The Netherlands
4 STI AIDS Netherlands, Amsterdam, The Netherlands
5 Department of General Practice, Amsterdam Medical Center/University of Amsterdam, Amsterdam, The Netherlands
6 Public Health Service Amsterdam, Amsterdam, The Netherlands
7 Public Health Service Rotterdam Rijnmond, Rotterdam, The Netherlands
8 Department of Public Health, Erasmus Medical Center, Rotterdam, The Netherlands
9 Public Health Service Southern Limburg, The Netherlands
10 School of Public Health and Primary Care (CAPHRI), Maastricht University Medical Centre (MUMC+), Maastricht, The Netherlands
11 Julius Center for Health Sciences and Primary Care, University Medical Center Utrecht, Utrecht, The Netherlands

Table S1: Important parameter values for the sexual network, and all parameter values for Chlamydia transmission used in the simulations. Parameters relating to the sexual network on the national level are taken from [1] (which lists all parameters used in detail, and includes the simulation code). Furthermore, all parameters that differ between the sexual network model on the national and the urban level are listed here. Incubation time and the duration of infection for untreated Chlamydia were taken from [2,3], and the fraction of asymptomatic infections was estimated by an expert panel of dutch GPs and STI center coordinators.

| | |
|---|---|
| **Population size:** | 50,000 individuals age 13-64, uniformly distributed |
| **Mean duration of partnership**<br>short-term<br>medium-term<br>long-term | <br>13⅓ days<br>200 days<br>5882 days (16.1 years) |
| **Model timestep**<br>Sexual network model – partner formation process<br>Ct transmission model | <br>4 days<br>1 day |
| **Number of partnerships:**<br>On the national level:<br>moderate men – moderate women<br>core men – core women<br>core men – moderate women<br>moderate men – core women<br><br>On the urban level:<br>moderate men – moderate women<br>core men – core women<br>core men – moderate women<br>moderate men – core women | <br><br>35.5%<br>0.3%<br>3.3%<br>1%<br><br><br>34%<br>2%<br>3%<br>2% |
| **Fraction of the population that will have a period of core-group behaviour during their life:**<br>On the national level<br>On the urban level | <br>5% women, 7% women<br>12% women, 12% men |
| **Onset of sexual availability (OSA)**<br>On the national level<br>On the urban level<br>Women<br>Men | <br>13 years + Gamma (k=1.2, theta=0.2) years<br><br>13 years + Gamma (k=1.2, theta=0.2) years<br>13 years + Gamma (k=1.2, theta=0.24) years |
| **Onset of core-group behaviour (OC)**<br>On the national level<br>On the urban level | <br>See Schmid & Kretzschmar, 2012<br>Idem to national level. |
| **Duration of core-group behavior (DC)**<br>On the national level<br>On the urban level | <br>See Schmid & Kretzschmar, 2012<br>Idem to national level. |
| **Maximum number of concurrent partnerships, for core-group individuals (i.e. 'maximum capacity')**<br>On the national level<br>Women<br>Men<br><br>On the urban level<br>Women<br>Men | <br><br>3 + DC / (0.14 * OC)<br>3 + DC / (0.28 * OC)<br><br><br>2 + DC / (0.08 * OC)<br>2 + DC / (0.16 * OC) |
| ***Chlamydia* Infection**<br>Incubation time<br>Fraction asymptomatic<br>Women<br>Men<br>Mean duration untreated *Ct* | <br>14 days<br><br>70%<br>50%<br>433 days |
| **Transmission rate *Chlamydia***<br>Main model<br>High treatment failure scenario | <br>0.765% per day and per relationship.<br>0.75% per day and per relationship. |

Figure S1: Effect of CSI on population prevalence of asymptomatic Chlamydia infections in men (solid lines) and women (dashed lines) for the baseline scenario, CSI screening and screening with a higher treatment failure rate of 8% (A) on national level and (B) in urbanized areas. In order to keep the baseline Chlamydia prevalence in the high treatment failure scenario at the same level as the baseline Chlamydia prevalence of the main model, the daily transmission chance of Chlamydia was reduced from 0.00765 to 0.0075.

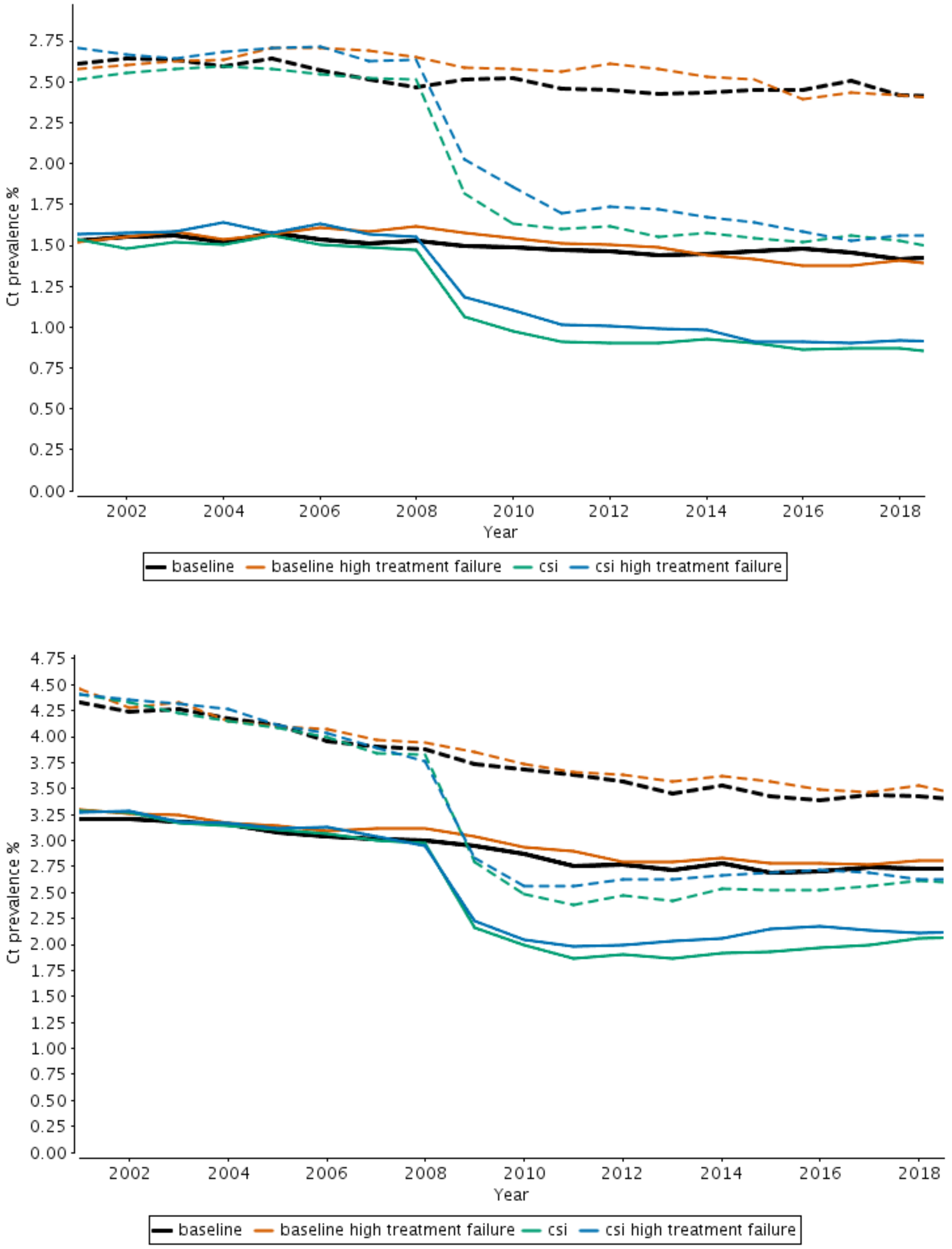

## References

1. Schmid BV, Kretzschmar M (2012) Determinants of sexual network structure and their impact on cumulative network measures. PLoS Comput Biol 8: e1002470.
2. Althaus CL, Turner KM, Schmid BV, Heijne JC, Kretzschmar M, et al. (2012) Transmission of Chlamydia trachomatis through sexual partnerships: a comparison between three individual-based models and empirical data. J R Soc Interface 9: 136-146.
3. Althaus CL, Heijne JC, Roellin A, Low N (2010) Transmission dynamics of Chlamydia trachomatis affect the impact of screening programmes. Epidemics 2: 123–131.
